# Does the visual system affect the learning curve of the Otosurgeon? A cadaveric study comparing microscopy vs exoscopy

**DOI:** 10.1007/s00405-025-09300-4

**Published:** 2025-03-26

**Authors:** Gabriele Testa, Carlo Conti, Isabelle Dohin, Mara Arcuri, Claudia Lodovica Modesti, Elisa Marazzi, Barbara Buffoli, Rita Rezzani, Davide Mattavelli, Silvia Zorzi, Daniele Borsetto, Michele Tomasoni, Vittorio Rampinelli, Cesare Piazza

**Affiliations:** 1 Department of Medical and Surgical Specialties, Radiological Sciences, and Public Health, School of Medicine, University of Brescia, Brescia, Italy; 2 Unit of Otorhinolaryngology-Head and Neck Surgery, ASST Spedali Civili of Brescia, 25123 Brescia, Italy; 3 Department of Pediatric Otorhinolaryngology, Spedali Civili, Piazza Spedali Civili, 1, 25100 Brescia, Italy; 4 Ear Nose and Throat (ENT), Department of Surgical Sciences, Dentistry, Gynecology and Pediatrics, University of Verona, Verona, Italy; 5 Section of Anatomy and Physiopathology, Department of Clinical and Experimental Sciences, University of Brescia, Brescia, Italy; 6 Department of ENT Surgery, Addenbrookes Hospital, Cambridge University Hospitals NHS Foundation Trust, Cambridge, UK

**Keywords:** Exoscope, Microscope, 3D, Temporal bone, Cadaveric dissection, Surgical training, Ear surgery, Cochlear implantation

## Abstract

**Purpose:**

Optical magnification is crucial in ear surgery, ensuring the precise identification of anatomical structures. Traditionally, microscopes have been the standard due to their magnification and stereoscopic capabilities. However, the introduction of exoscopes has introduced new possibilities, particularly in ergonomics, teaching, collaboration, and surgical training. This cadaveric study aimed to evaluate the feasibility, effectiveness, and trainee performance when using the exoscope in ear dissection and to compare it with the traditional microscope.

**Methods:**

This study involved 10 non-expert medical trainees who undertook a series of surgical tasks on cadaveric specimens using both the microscope and exoscope. The tasks included different surgical approach simulations and exercises. NASA Task Load Index and a Visual Analog Scale questionnaires were administered to assess participants' subjective experiences with each instrument.

**Results:**

All participants successfully completed the assigned tasks with both the microscope and exoscope. While there were no significant differences in the timing of surgical steps between the two instruments, participants perceived the microscope as less physically demanding and temporally taxing, while the exoscope excelled in structural identification and offered benefits for teaching and collaboration.

**Conclusions:**

The choice between microscope and exoscope should be guided by the specific surgical demands, educational context, and preferences of the team. While the microscope excels in flexibility, the exoscope provides advantages in structural identification and collaborative learning, making it a valuable tool in ear surgery. This study contributes valuable insights for otological surgeons and educators to optimize surgical outcomes and learning experiences of trainees.

**Supplementary Information:**

The online version contains supplementary material available at 10.1007/s00405-025-09300-4.

## Introduction

Optical magnification for identification of anatomical structures is essential to perform ear surgery safely and successfully [1]. Since the 1920s, the traditional microscope has been the cornerstone of otologic and neurotologic surgery, offering magnified and, through binocular vision, stereoscopic views of the surgical field [2]. Over the past two decades, endoscopic ear surgery has gained prominence, either as an exclusive approach or in combination with the microscope [3, 4]. The endoscope provides a heads-up approach, improving ergonomics and allowing the surgical team and trainees to view the procedure on a screen, enhancing teaching potential, and offers unparalleled wide-angle view and detection of anatomical details through narrow surgical corridors [5]. However, its use presents challenges: the instrument occupies space, creating steric hindrance and its use requires a very steep learning curve, especially to adapt to one-handed surgery and to its lack of true stereoscopic depth perception [6]. In recent years, the development of 4 K high-definition and 3-dimensional video telescopes, i.e. the exoscope, has evolved due to technological advancements. This device lies exterior to the body and generates stereoscopic images on high-resolution screens, thus allowing the surgeon to operate with two hands in ergonomic posture [7, 8]. The 3D exoscope has been recently employed in several otolaryngological procedures [9–12], including otoneurologic surgery, involving the middle ear, mastoid, and stapes surgery [8, 13], vestibular schwannomas and lateral skull base surgery [14], and cochlear implant surgery [1, 15]. Ergonomics and its intuitive nature are the advantages most commonly described by several authors reporting their preliminary experiences [1, 13, 16]. Moreover, as the endoscope, since assistants or students share the same image as the surgeon on the screen, the exoscope has greater teaching potential compared to other visual systems, and in particular the microscope [17].

Regardless of the visual modality used, operative dissection of cadaveric temporal bone has a crucial role in otologic surgical training to develop improved hand–eye coordination, learn command of fine motor skills, and obtain thorough anatomical knowledge under magnified vision [18].

To the best of our knowledge, to date, an objective and subjective comparison between microscope and exoscope as visualization tools in otologic surgery within the cadaver training setting has never been reported. The aim of this cadaveric study is therefore to assess feasibility, effectiveness, advantages, and disadvantages of using an exoscope in temporal bone dissection. Moreover, the performances of medical trainees in otologic procedures during cadaveric dissection performed with microscope and exoscope have been compared. Given its specific visualization characteristics, the endoscope was not included in our evaluation.

## Materials and methods

A group of 10 early year residents with limited experience in otologic and/or microscopic surgery was enrolled. Ten fixed-formalin adult human heads (MedCure Inc., Portland, Oregon, USA) were dissected bilaterally in the Anatomical Training Center of the University of Brescia. The specimens were placed on a holder mimicking the position of the patient’s head during otological surgery.

**Fig. 1:**
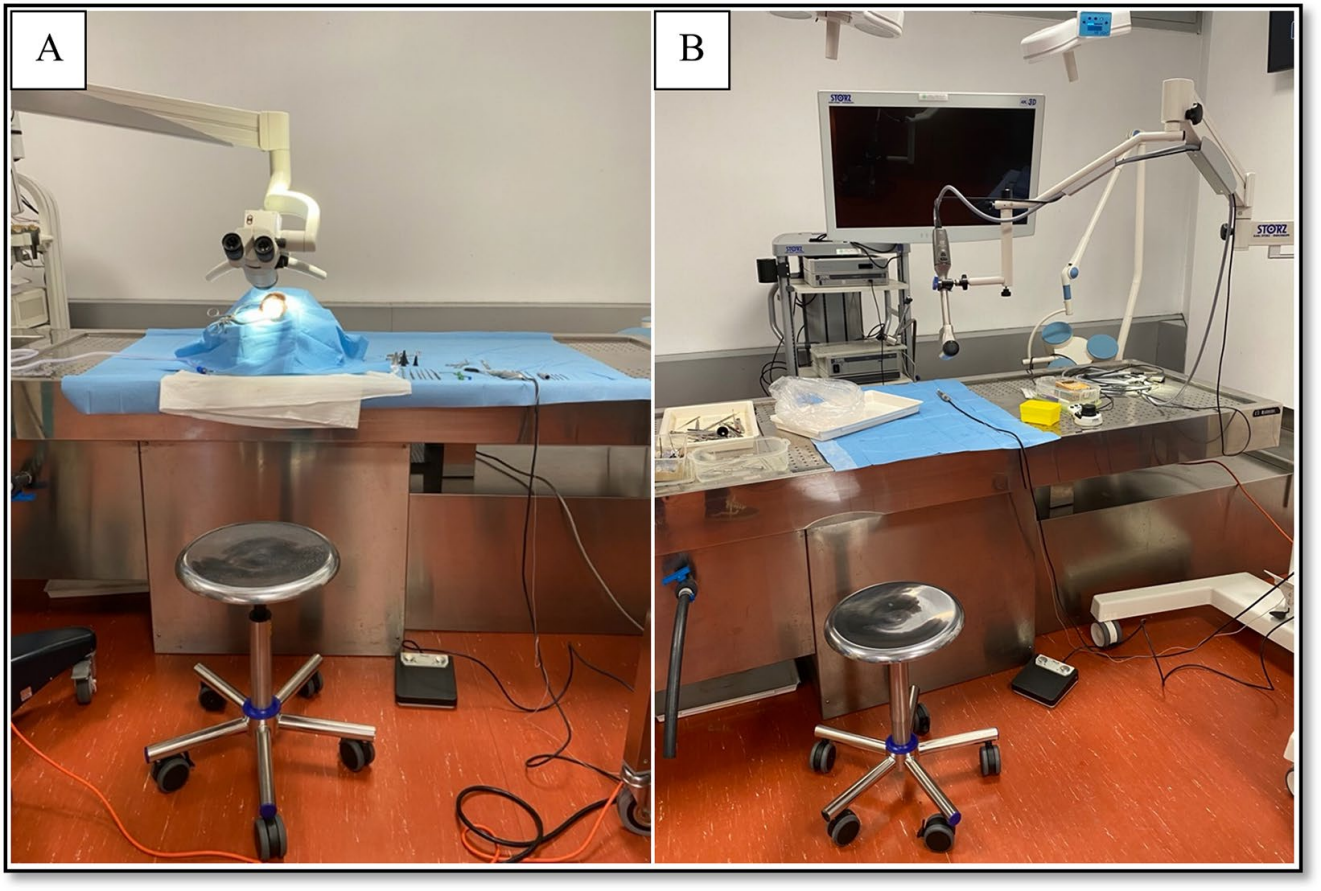
Microscope (**A**) and xoscope Vitom 3D exoscope (**B**) settings

A sequence of surgical tasks was designed, including surgical approaches simulations and abstract tasks to be performed with an exoscopic 3D system (VITOM 3D exoscope, mounted on VERSACRANE™ holding arm and a full HD 3D monitor, Karl Storz, Tuttlingen, Germany) and a microscope (ENT surgical microscope Leica M320 F12 with multifocal objective lens with variable working distance of 200 mm to 300 mm, with 5-step magnification changer—6.4 / 10 / 16 / 25 / 40x—and integrated full HD camera). Figure 1 shows pictures of the microscopic and exoscopic settings.

**Fig. 2:**
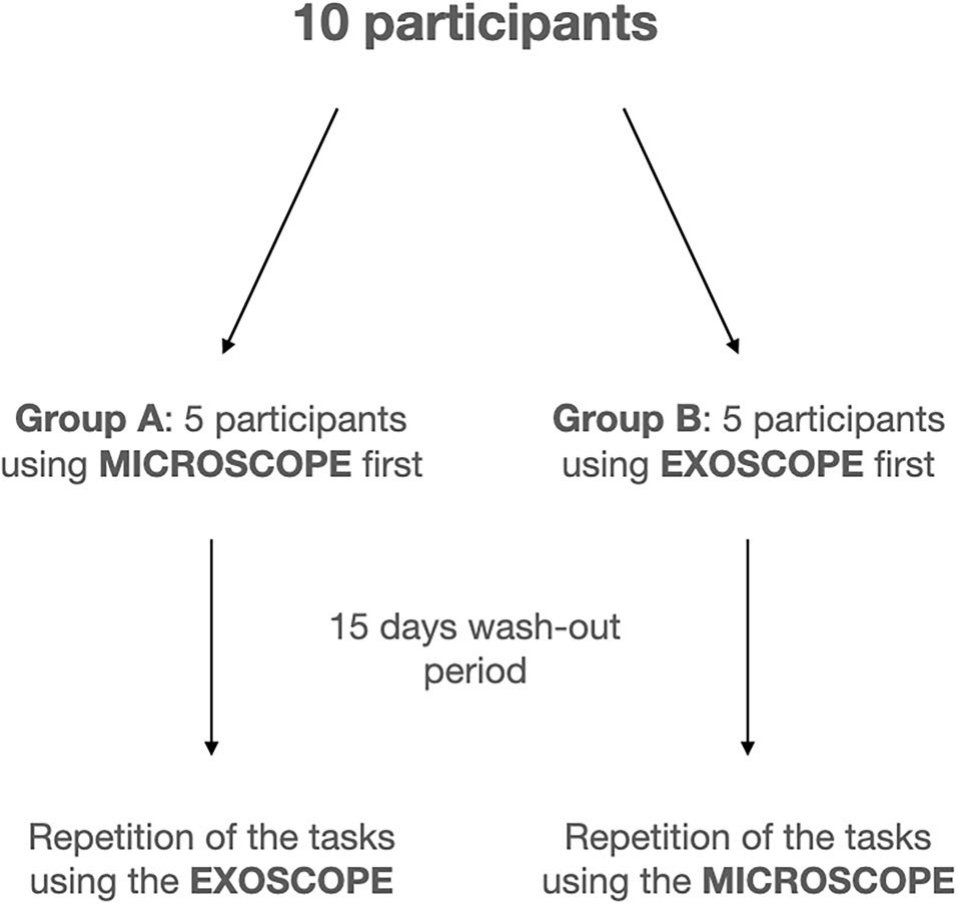
Scheme of the study protocol

Each participant was assigned to a specimen, 5 (group A) had to complete the surgical tasks using the microscope on the first side and the exoscope on the second one, while the remaining 5 (group B) had to operate with the exoscope before using the microscope. A washout time of 15 days was set between the 2 executions (Fig. 2).

Participants were asked to perform a retroauricolar and a transcanal surgical approach on the cadaver, following a step-by-step dissection the passages of which are reported in Table 1.

**Table 1:** Step-by-step passages of surgical approaches

Surgical approaches	Tasks
Retroauricolar approach	• Retroauricolar incision • Palva’s flap elevation • Mastoidectomy • Posterior tympanotomy • Drilling of the round window niche • Insertion of the cochlear implant electrode into the scala tympani
Transcanal approach	• Tympanomeatal flap elevation • Atticotomy • Incudostapedial joint disarticulation • Removal of the stapes superstructure • Platinotomy • Stapes prosthesis positioning

**Fig. 3:**
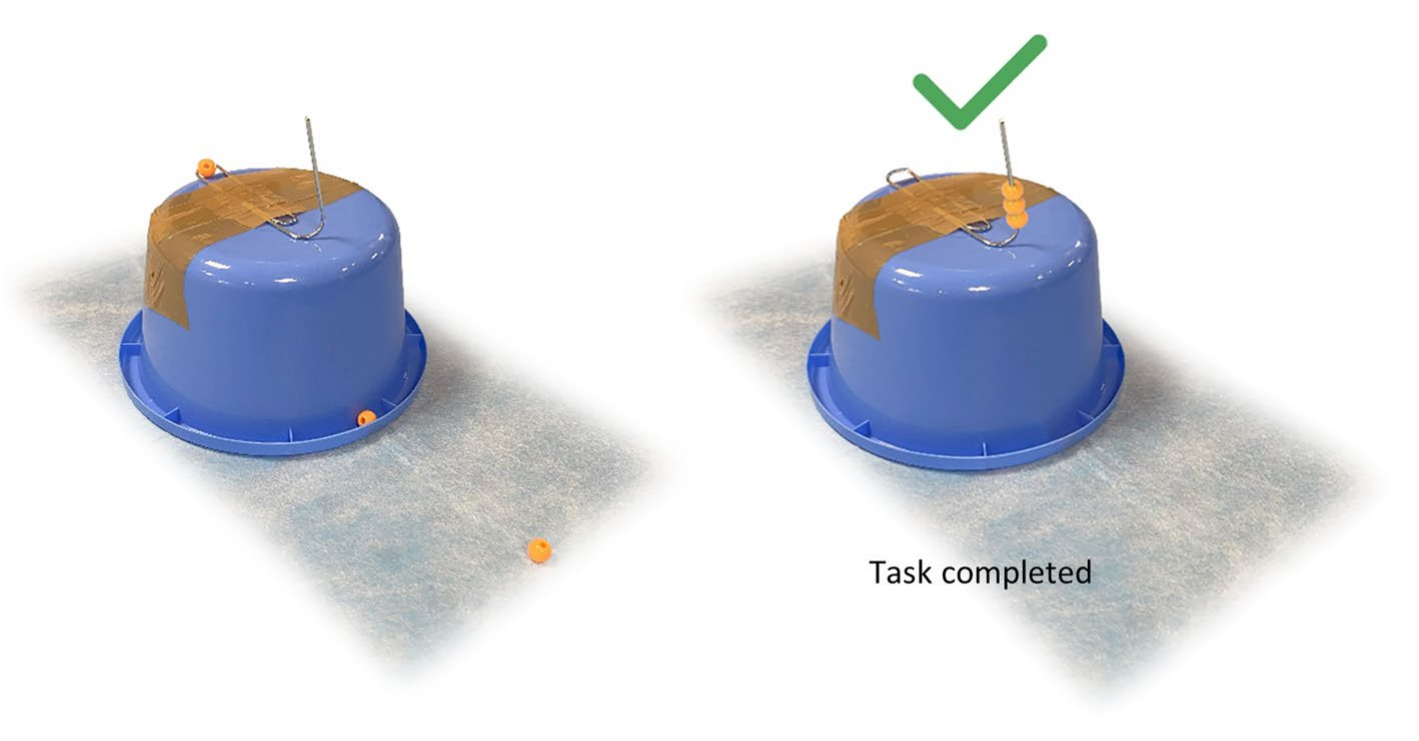
Pearl-piling task set: three 4 mm pierced pearls placed in 3 different spots and a plastic support with a secured needle set from a paper clip; the participant is asked to pile the pearls through their holes on the needle with Hartmann ear forceps

**Fig. 4:**
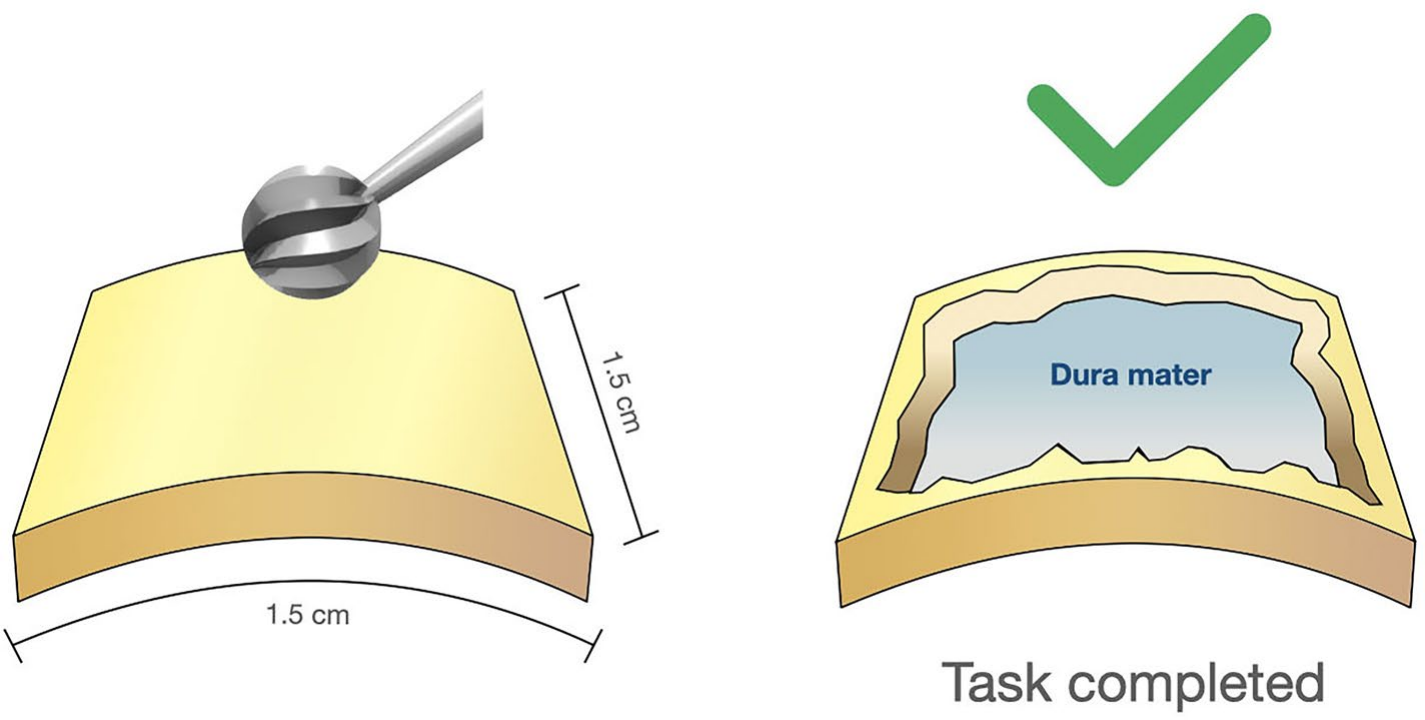
Calvaria task: the participant is asked to drill with a 6 mm cutting bur a 1.5 × 1.5 cm^2^ area of the calvarian parietal bone until the dura mater is reached and uniformly exposed on the entire surface drilled, without damaging the dura itself

Moreover, two additional tasks were designed. The first consisted in taking three beads placed in different spots and pile them on a needle (Fig. 3), and the second in drilling a 1.5 cm^2^ of calvarial bone until exposure of the dura was obtained (Fig. 4). Participants were shown explanatory videos before the start of each procedure.

The following steps/task were timed: setting of the instrument (positioning of the exoscope or microscope and focus setting); incudo-stapedial joint disarticulation; platinotomy; positioning of the teflon stapes piston prosthesis (Grace Medical Inc, Memphis, USA) in the platinotomy anchoring it to the long process of the incus; drilling of a 1.5 × 1.5 cm area of calvaria until reaching the dura; insertion of 3 plastic pearls located in 3 different spots of the operating field on a needle; insertion of the Flex28 cochlear implant electrode from MED-EL, Innsbruck, Austria.

After the completion of the tasks, each participant was asked to complete two questionnaires: NASA Task Load Index (TLX) questionnaire [19] (Appendix 1, supplementary material), an assessment tool that rates perceived workload based on 6 items (mental demand, physical demand, temporal demand, performance, effort and frustration) graded from 1 (very low) to 5 (very high), and a Visual Analog Scale from 1 to 10 (VAS 1–10) questionnaire, in which they expressed their judgment (on a scale from 1 to 10 in which 1 is the worst experience and 10 the best) on both the exoscope and the microscope, related to the instrument setting, handling, image quality, three-dimensionality of the image, and encumbrance of the device (Appendix 2, supplementary material).

The aim of the study was to evaluate the differences of the two visualization methods in terms of timing of surgical steps and the subjective feeling at the beginning of the learning curve. Statistical analysis of operative times and questionnaires scoring was carried out with a Mann–Whitney test. Statistical significance was set at *p* < 0.05.

## Results

### Surgical steps

All the participants successfully completed the tasks with both the microscope and exoscope. No significant anatomic variations or pathological findings were identified on the specimens. The median execution times under visual assistance with the exoscope and the microscope are reported in Table 2, while the detailed timings are reported in Appendix 3 (supplementary material)*.* There was no significant difference between group A and B in terms of time of execution of the surgical steps, and there was no significant difference in timing between the microscope and exoscope except for setting and the pearl-piling task where the microscope had shorter times (Appendix 3, supplementary material).

**Table 2:** Median execution times of the timed tasks

Task	Microscope (IQR)	Exoscope (IQR)	Delta (exoscope—microscope)
Setting	6 s 500 ms (5 s 250 ms–8 s 500 ms)	10 s (9 s–11 s 500 ms)	3 s 500 ms
Incudo-stapedial joint disarticulation	14 s 500 ms (10 s 750 ms–15 s 750 ms)	14 s 500 ms (12 s 500 ms–16 s 750 ms)	0
Platinotomy	16 s 500 ms (15 s 250 ms–17 s)	18 s (13–22 s)	1 s 500 ms
Stapes prosthesis positiong	5 min 41 s 500 ms (3 min 26 s 750 ms–6 min 50 s 750 ms)	6 min 31 s 500 ms (5 min 39 s 750 ms–7 min)	50 s
Calvarial drilling	1 min 59 s (1 min 49 s–2 min 12 s 250 ms)	1 min 58 s (1 min 27 s 500 ms–2 min 12 s 500 ms)	−1 s
Pearl piling on the needle	26 s 500 ms (24 s–29 s 250 ms)	1 min 50 s (1 min 40 s–2 min 750 ms)	1 min 23 s 500 ms
Implant electrode insertion	6 min 41 s (5 min 54 s 500 ms–7 min 29 s 250 ms)	6 min 32 s (5 min 36 s 250 ms–7 min 21 s 250 ms)	−9 s

### Nasa TLX

According to the participants, using the microscope was equally mentally [median 2.5 (IQR: 2–3) vs median 3.0 (IQR: 3–3); *p* = 0.12] and less physically [median 2 (2–3) vs median 3.5 (3–4); *p* = 0.003], and temporally demanding [median 2 (1–2) vs mean 3 (3–3); *p* = 0.001] compared to the exoscopic visualization system. The efforts required [median 2.0 (1,25–2,75) vs median 3.5 (3–4); *p* = 0.0015] and perceived frustration [median 2 (1–2) vs median 3.5 (3–4); *p* = 0.0004] were also lower during microscopic visual assistance. Detailed NASA TLX scores are reported in Appendix 4 (supplementary material). There was no significant difference between the scores reported by groups A and B.

### VAS 1–10

Concerning VAS scores reported by participants, no significant difference between groups A and B was found. In Appendix 5 (supplementary material), the participants VAS scores are reported. Handling [median 9 (9–10) vs median 6.5 (6–7); *p* = 0.00018] and three-dimensionality [median 9 (9–10) vs median 8.0 (7.25–8); *p* = 0.007] were significantly perceived as better for the microscope, while quality of images [median 9.5 (9–10) vs median 8 (8–8.75); *p* = 0.0015] and advantages of limited encumbrance [median 9 (8–9) vs median 7.5 (7–8); *p* = 0.02] were considered as benefits of the exoscopic visualization system.

## Discussion

Over the last years, use of the exoscope has gained increasing interest in head and neck surgery, including otology and lateral skull base procedures [20]. As reported in the systematic review by Ferlito et al. [20], this device has been shown to be effective in the treatment of chronic pathologies of the middle ear and several tumor types of the external and middle ear, and to perform cochlear implant surgery [1, 8, 15, 16, 21–23].

The exoscope is also emerging as a valuable tool in surgical training. In 2020, De Virgilio et al. published a paper demonstrating the feasibility of exoscope-based microsurgery training in a group of 22 medical students, who were asked to perform 4 exercises assessing basic microsurgical skills [24]. The study demonstrated that the exoscope was promising in terms of technological quality and technical feasibility. Two years later, the same group conducted a study to evaluate the use of the 3D-4 K exoscope in temporal bone dissection and its potential for teaching purposes [25]. It involved 6 exercises performed by surgeons with varying levels of expertise and evaluated their outcomes using the Melbourne Mastoidectomy Scale, a novel scale developed specifically for evaluating cortical mastoidectomy [26]. The medium scores for the novice, intermediate, and expert surgeons were comparable for the same categories of expertise reported by the scale validation group [26], suggesting that the exoscope is adequate for safe and effective mastoidectomy and exhibited great potential for educational use [25].

In this context, to our knowledge, our study is the first on temporal bone dissection and otologic procedures, directly comparing the 3D exoscope and microscope, focusing both on objective operative outcomes and subjective assessment of trainees. All participants successfully completed the assigned surgical tasks using both the microscope and exoscope, confirming the feasibility of these systems in otologic surgery even for individuals with limited experience. No significant difference was found in the timing of surgical steps between participants who used the exoscope first and those that used the microscope first, suggesting that the order of exposure to the two visual systems did not significantly impact the participants' ability to perform the tasks efficiently. This is a valuable insight, as it implies that the exoscope could be integrated into training without adversely affecting the learning process, allowing trainees to adapt to its use without sacrificing surgical efficiency.

However, it is important to note that there were specific surgical steps (setting and pear-piling task) where the microscope outperformed the exoscope in terms of operative time. This could be attributed to the trainees' familiarity, although limited, with the microscope, which might result in more precise and quicker maneuvering, considering its daily use in office. Over time, with additional exposure and practice, it is possible that the differences in timing between the two instruments might diminish. Subjective assessment of the exoscope and microscope provided valuable insights into the trainees' experiences, revealing the advantages and trade-offs that the exoscope has over the traditional microsocope.

### Exoscopic vs microscopic vision quality

**Fig. 5:**
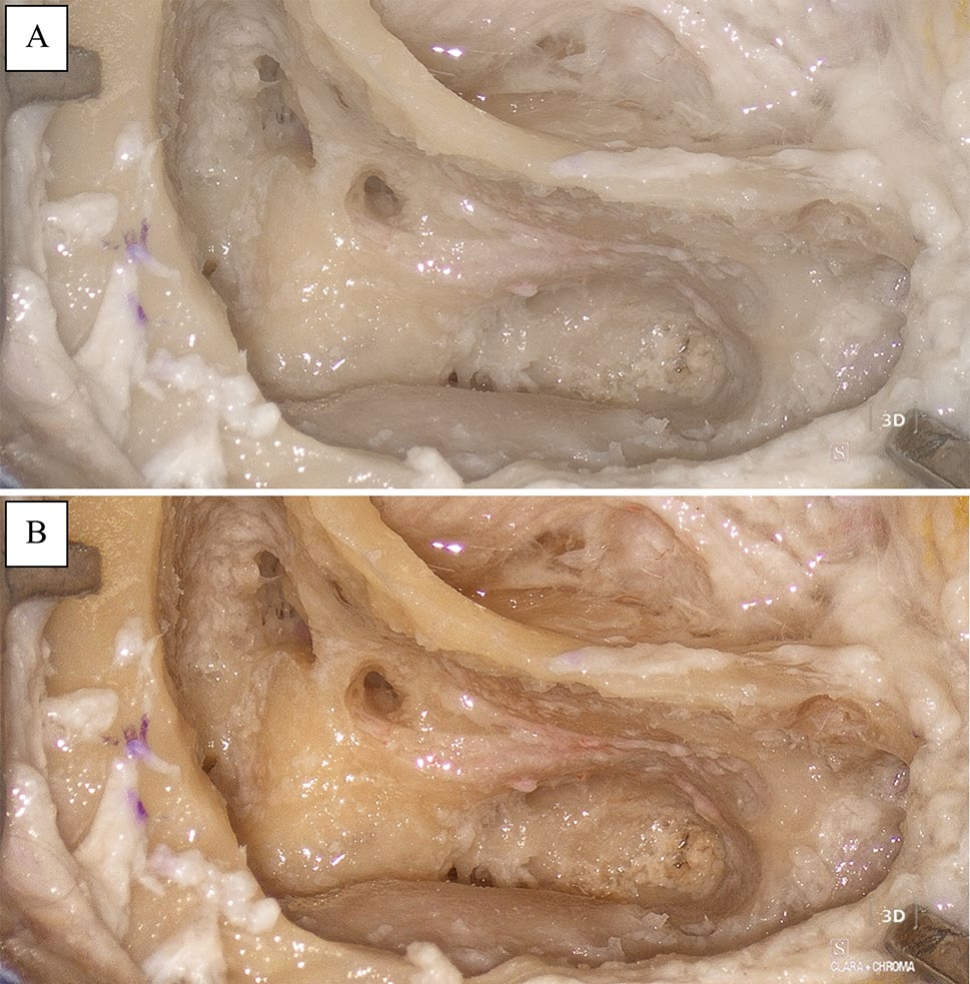
Differences in visualization of structures with no filter (**A**) and Clara + Chroma filter (**B**) of the Vitom 3D

Image quality provided by the exoscope was judged better by VAS, which is probably due to the remarkable ability of exoscope filters to pinpoint critical structures such as the facial nerve, dura, and sigmoid sinus (Fig. 5). As also highlighted in the study by Rubini et al. [23], this outcome underscores the role of exoscopic visualization as a valuable tool in cases where the utmost precision is paramount, since surgeons can leverage these filters to mitigate the risk of inadvertent damage to delicate anatomical structures.

However, exoscopic image quality presents two major issues that are described in several studies and are confirmed by ours. The first is the reduction of image quality in extreme magnification, resulting in worse definition, contrast, and brightness of the image. Even if this problem can be partially overcome by moving the exoscope head closer to the surgical field as stated by Colombo et al. [1], the worsening of image quality due to its digital processing can lead to severe difficulties in distinguishing structures of the same brightness (for example dura and cholesteatoma), thus potentially increasing the risk of having a complication at high magnifications [8]. However, it must be clarified that the study was conducted using the VITOM 3D, which is not specifically adapted to ear surgery yet, since it still has a suboptimal depth of field. While this remains a challenge, possible future advancements in exoscopic technology could help mitigate this limitation, improving depth perception and adaptability for this kind of surgery. A second issue is represented by the reduction of visibility in narrow, deep, and tortuous surgical corridors such as the external auditory canal that prevent the light from fully strike the area of interest. In our study, there was no significant difference between timing of the tasks performed through the transcanal corridor between the microscope and exoscope, although Wierzbicka et al. had to convert two procedures to the microscope for the lack of visibility using the exoscope in a transcanal approach [21]. Moreover, Milanesi et al. concluded that transcanal approaches do not seem to grant sufficient visualization of the field with the exoscope [13]. In our study, the same issue was not encountered when visualizing through a posterior tympanotomy and there was no significant difference in the timing of electrode insertion between the microscope and exoscope. Our results are confirmed by the study of Tan et al. who reported no visualization issues in cochlear implant surgery with the exoscope [27]. A noteworthy limitation of the microscope, as highlighted in our study, is its inability to provide stereoscopic vision to those observing the surgery. This deficiency necessitates the use of an external camera for individuals to watch the dissection. Conversely, the exoscope excels by offering a stereoscopic view without the need for additional equipment a part from a pair of 3D-glasses. This streamlined approach enhances the overall efficiency of the surgical procedure.

### Handling and ergonomics

While exoscopic visualization has advantages in terms of structural identification, our study reveals that the microscope stands out for its adaptability in terms of altering the field of view. The ability to manipulate the vision and perspective in real-time can be crucial during surgery, particularly when navigating complex anatomical structures demanding rapid adjustments to the visual perspective. This aspect is highlighted by the subjective impressions expressed herein, in which handling was deemed to be easier using the microscope. The same perceptions were noted by Minoda er al. [8] and Zhang et al. [7], who reported that changing the visual field and refocusing to obtain the right 3D surgical vision was uncomfortable and difficult.

However, it must be specified that in our study we were equipped with a fully manual arm and that handling could have been improved by using a robotic arm (i.e. ARTip CRUISE, Karl Storz) which enables more precise and smoother movements of the exoscope.

The exoscope’s ability to promote better back posture during surgery is a finding that resonates with surgeons who understand the importance of ergonomics. Maintaining proper posture is not merely a matter of comfort; it directly impacts a surgeon's performance and longevity in the profession [28].

In our study, the microscope was also perceived as more physically demanding with respect to the exoscope. As mentioned by Ally et al., the Vitom 3D system is superior to the microscope, as it allows otologists to operate in the head-up position. [16]. Moreover, the monitor is oriented in the direction of the hands, thus facilitating movements and maintaining a more comfortable position of neck and spine [1, 13].

### Exoscope’s benefits for teaching and collaboration

One of the most interesting aspects mentioned in the literature is the superior performance of the exoscope when it comes to collaborative environments. The stereoscopic and high-quality images offered by the exoscope make it an invaluable tool for teaching and facilitating collaboration between the primary surgeon, assistants, and trainees, who all share the exact same view [1, 7]. Furthermore, this enhanced visibility serves to benefit expert surgeons who are instructing trainees, ensuring that they can effectively convey the nuances of the procedure. Moreover, for the same reasons, the exoscope would fit perfectly in a temporal bone dissection, allowing for a more shared experience and easier supervision by the tutor which would translate into more precise tutor’s advice and better understanding of anatomy and surgical steps [25].

### Clinical implications and future directions

Our results indicate that the exoscopic 3D system is a viable alternative to the traditional microscope in otologic surgery. It may offer advantages in terms of reduced encumbrance, use of vision filters, and educational purposes, which can contribute to the overall surgical experience. Additionally, the exoscope can be readily adopted by trainees without significantly affecting their operative efficiency.

For future research, it would be valuable to assess the performance of experienced ear surgeons when using the exoscope, since their feedback may differ from that of novice trainees. Additionally, it would be interesting to evaluate how the new visualization systems could affect the progression of the learning curve through longitudinal studies. Long-term studies could also explore the continued impact of instrument choice on operative times and clinical outcomes as trainees progress through their learning curve.

### Limitations

One of the greatest limitations, possibly biasing our results, is represented by the equipment used for the experiment, namely the microscope Leica M320 and the exoscope Vitom 3D by STORZ, since neither of them represents the benchmark technology for middle ear surgery. More powerful and effective microscopes are widely available and exoscope technology is evolving rapidly. Devices with better ergonomics, smoother handling, and a more suitable depth of field, are likely to be available soon. Moreover, the familiarity the participants have with the microscope considering the daily in-office use may represent an additional limitation and possible source of bias.

## Conclusions

Our study underscores the importance of a nuanced approach when choosing between exoscopic and microscopic visualization in otologic surgery, considering the specific surgical task, educational context, and available technology. Based on the equipment tested, despite its inherent limitations, exoscopic visualization excelled in structural identification and collaborative learning, while the microscope offered greater flexibility for dynamic field adjustments. However, as technology evolves, many of the limitations identified may become less relevant, with newer exoscopes addressing depth perception and adaptability. These insights could help surgeons and educators make informed decisions while considering the capabilities of the tools available in their setting.

## Supplementary Information

Below is the link to the electronic supplementary material.

Supplementary file1 (DOCX 38 kb)

## Data Availability

The data that support the findings of this study are available from the corresponding author, VR, upon reasonable request.
