# Supplementary material for: Does the visual system affect the learning curve of the Otosurgeon? A cadaveric study comparing microscopy vs exoscopy
Source: Eur Arch Otorhinolaryngol. 2025 Mar 26;282(8):3941–8. doi: 10.1007/s00405-025-09300-4 (PMC12399444; doi:10.1007/s00405-025-09300-4)
Supplement: Supplementary file 1 — Supplementary file1 (DOCX 38 kb) [file 405_2025_9300_MOESM1_ESM.docx]

**Supplementary material - Appendix**

**Appendix 1:** The NASA TLS questionnaire (Hart and Staveland, 1988) used in the current study.

You will be presented with a set of evaluation scales for exoscope and endoscope. For each of the six scales, evaluate the activity you have recently done by placing a cross on the position of the ladder that corresponds to your experience. The scale provides a score from 1 to 5 in which 1 = very low and 5 = very high. Carefully consider your answers when distinguishing between the different conditions of the task and consider each scale individually.

**A. MENTAL DEMAND**

How mentally demanding was the task?

| Exoscope |  |  |  |  |  |
| --- | --- | --- | --- | --- | --- |
| Microscope |  |  |  |  |  |
|  | VERY LOW | LOW | NEUTRAL | HIGH | VERY HIGH |

**B. PHYSICAL DEMAND**

How physically demanding was the task?

| Exoscope |  |  |  |  |  |
| --- | --- | --- | --- | --- | --- |
| Microscope |  |  |  |  |  |
|  | VERY LOW | LOW | NEUTRAL | HIGH | VERY HIGH |

**C. TEMPORAL DEMAND**

How hurried or rushed was the pace of the task?

| Exoscope |  |  |  |  |  |
| --- | --- | --- | --- | --- | --- |
| Microscope |  |  |  |  |  |
|  | VERY LOW | LOW | NEUTRAL | HIGH | VERY HIGH |

**D. PERFORMANCE**

How successful were you in accomplishing what you were asked to do?

| Exoscope |  |  |  |  |  |
| --- | --- | --- | --- | --- | --- |
| Microscope |  |  |  |  |  |
|  | VERY LOW | LOW | NEUTRAL | HIGH | VERY HIGH |

**E. EFFORT**

How hard did you have to work to accomplish your level of performance?

| Exoscope |  |  |  |  |  |
| --- | --- | --- | --- | --- | --- |
| Microscope |  |  |  |  |  |
|  | VERY LOW | LOW | NEUTRAL | HIGH | VERY HIGH |

**F. FRUSTRATION**

How insecure, discouraged, irritated, stressed and annoyed were you performing the task?

| Exoscope |  |  |  |  |  |
| --- | --- | --- | --- | --- | --- |
| Microscope |  |  |  |  |  |
|  | VERY LOW | LOW | NEUTRAL | HIGH | VERY HIGH |

**Appendix 2:** The VAS 1-10 questionnaire used in the study.

Score from 1 to 10 (1=worst experience; 10=best experience)

|  | **EXOSCOPE** | **ENDOSCOPE** |
| --- | --- | --- |
| Setting |  |  |
| Handling |  |  |
| Quality of images |  |  |
| Three-dimensionality |  |  |
| Advantages of limited encumbrance |  |  |

**Appendix 3**: Times of executions of the surgical tasks of group A (participants 1-5) and group B (participants 6-10).

| **Participant** | **Setting** | | **Electrode Insertion** | | **Incudo-stapedial joint disarticualtion** | | **Platinotomy** | | **Stapes prosthesis placement** | | **Calvarial drilling** | | **Pearl piling on the needle** | |
| --- | --- | --- | --- | --- | --- | --- | --- | --- | --- | --- | --- | --- | --- | --- |
|  | Microscope | Exoscope | Microscope | Exoscope | Microscope | Exoscope | Microscope | Exoscope | Microscope | Exoscope | Microscope | Exoscope | Microscope | Exoscope |
| **1** | 7s | 10s | 4min 56s | 5min 13s | 8s | 8s | 13s | 13s | 3min 34s | 4min 6s | 2min 18s | 1min 27s | 26s | 1min 23s |
| **2** | 5s | 10s | 6min 37s | 5min 55s | 14s | 14s | 16s | 18s | 5min 43s | 6min 3s | 1min 48s | 1min 49s | 27s | 1min 34s |
| **3** | 9s | 12s | 5min 45s | 5min 30s | 17s | 20s | 20s | 20s | 6min 32s | 6min 45s | 2min 2s | 2min 11s | 24s | 1min 45s |
| **4** | 6s | 12s | 7min 13s | 7min 37s | 13s | 15s | 17s | 20s | 5min 38s | 7min 24s | 1min 23s | 2min 23s | 30s | 1min 57s |
| **5** | 5s | 8s | 6min 23s | 6min 30s | 20s | 19s | 20s | 22s | 7min 23s | 6min 45s | 1min 52s | 1min 34s | 24s | 2min 13s |
| **6** | 7s | 9s | 5min 32s | 5min 18s | 15s | 17s | 17s | 18s | 6min 23s | 6min 18s | 1min 35s | 1min 48s | 30s | 2min 2s |
| **7** | 5s | 8s | 7min 24s | 7min 30s | 16s | 14s | 15s | 15s | 6min 57s | 7min 13s | 2min 4s | 2min 15s | 35s | 2min 5s |
| **8** | 6s | 9s | 6min 45s | 6min 34s | 15s | 16s | 16s | 17s | 7min 14s | 7min 5s | 2min 15s | 2min 7s | 23s | 1min 43s |
| **9** | 10s | 10s | 7min 18s | 7min 24s | 9s | 11s | 13s | 15s | 4min 45s | 4min 40s | 1min 17s | 1min 25s | 24s | 1min 55s |
| **10** | 9s | 12s | 6min 58s | 7min 13s | 10s | 12s | 17s | 18s | 5min 40s | 5min 32s | 1min 56s | 2min 13s | 27s | 1min 29s |
|  | p-value: 0,0041 | | p-value: 0,97 | | p-value: 0,67 | | p-value: 0,17 | | p-value: 0,62 | | p-value: 0,15 | | p-value: 0,0002 | |

**Appendix 4**: NASA TLS scores from group A (1-5) and group B (6-10).

|  | **NASA TLS (1= very low, 5= very high)** | | | | | | | | | | | |
| --- | --- | --- | --- | --- | --- | --- | --- | --- | --- | --- | --- | --- |
| **Participant** | **Mental demand** | | **Physical demand** | | **Temporal demand** | | **Performance** | | **Effort** | | **Frustration** | |
|  | Microscope | Exoscope | Microscope | Exoscope | Microscope | Exoscope | Microscope | Exoscope | Microscope | Exoscope | Microscope | Exoscope |
| **1** | 3 | 4 | 2 | 4 | 1 | 3 | 4 | 4 | 1 | 3 | 2 | 4 |
| **2** | 2 | 2 | 3 | 4 | 2 | 3 | 3 | 4 | 3 | 4 | 1 | 3 |
| **3** | 3 | 3 | 1 | 3 | 1 | 3 | 4 | 3 | 2 | 3 | 2 | 4 |
| **4** | 3 | 3 | 3 | 3 | 2 | 4 | 4 | 2 | 2 | 4 | 1 | 2 |
| **5** | 2 | 3 | 2 | 4 | 1 | 3 | 3 | 3 | 1 | 3 | 2 | 4 |
| **6** | 3 | 4 | 2 | 4 | 3 | 3 | 4 | 4 | 3 | 4 | 2 | 3 |
| **7** | 1 | 3 | 3 | 3 | 2 | 3 | 3 | 4 | 2 | 4 | 2 | 3 |
| **8** | 2 | 3 | 2 | 3 | 2 | 2 | 4 | 3 | 2 | 3 | 1 | 4 |
| **9** | 2 | 2 | 2 | 4 | 1 | 4 | 4 | 2 | 1 | 4 | 1 | 3 |
| **10** | 3 | 3 | 3 | 3 | 2 | 3 | 4 | 2 | 3 | 3 | 2 | 4 |
|  | p-value: 0,12 | | p-value: 0,003 | | p-value: 0,001 | | p-value: 0,15 | | p-value: 0,0015 | | p-value: 0,0004 | |

**Appendix 5**: VAS scores reported by group A (1-5) and group B (6-10).

|  | **VAS scale 1-10 (1= worst experience 10= best experience)** | | | | | | | | | |
| --- | --- | --- | --- | --- | --- | --- | --- | --- | --- | --- |
| **Participant** | **Setting** | | **Handling** | | **Quality of images** | | **Three-Dimensionality** | | **Advantages of limited encumbrance** | |
|  | Microscope | Exoscope | Microscope | Exoscope | Microscope | Exoscope | Microscope | Exoscope | Microscope | Exoscope |
| **1** | 8 | 10 | 10 | 7 | 8 | 10 | 10 | 8 | 7 | 9 |
| **2** | 9 | 9 | 9 | 6 | 8 | 9 | 9 | 7 | 7 | 9 |
| **3** | 8 | 10 | 10 | 7 | 7 | 9 | 9 | 9 | 8 | 8 |
| **4** | 10 | 10 | 9 | 7 | 9 | 10 | 10 | 8 | 6 | 9 |
| **5** | 10 | 7 | 9 | 6 | 8 | 9 | 10 | 8 | 7 | 7 |
| **6** | 7 | 10 | 10 | 6 | 9 | 10 | 9 | 7 | 8 | 9 |
| **7** | 10 | 10 | 9 | 7 | 7 | 9 | 9 | 8 | 8 | 8 |
| **8** | 8 | 8 | 9 | 6 | 8 | 10 | 10 | 7 | 9 | 9 |
| **9** | 10 | 10 | 9 | 5 | 8 | 9 | 8 | 8 | 8 | 9 |
| **10** | 8 | 10 | 10 | 8 | 9 | 10 | 9 | 10 | 7 | 8 |
|  | p-value: 0,27 | | p-value: 0,00018 | | p-value: 0,00152 | | p-value: 0,007 | | p-value: 0,02 | |
